# Embolization management of post-traumatic hepatic pseudoaneurysm: A compelling case report

**DOI:** 10.1016/j.radcr.2025.01.050

**Published:** 2025-03-09

**Authors:** Hiba Ben Hassine, Ahmed Hadj Tayeb, Maissa Jalleli, Sadek Ben Jabra, Jamel Saad, Faouzi Noomen

**Affiliations:** a Department of Visceral Surgery, Fattouma Bourguiba Hospital, Monastir, Tunisia; b Department of Radiology, Fattouma Bourguiba Hospital, Monastir, Tunisia

**Keywords:** Embolization, Hepatic artery, Post-traumatic hepatic pseudo aneurysm, Case report

## Abstract

Pseudoaneurysm of the hepatic artery, first described by Sandblom in 1948, is an uncommon complication of hepatic trauma. Since then, numerous cases have been documented. Post-traumatic hepatic pseudoaneurysm of the hepatic artery is a rare complication, occurring in approximately 1.2% of patients with traumatic liver injury.

We report the case of a 25-year-old patient with a history of Behçet's disease, treated with colchicine, who sustained a traffic accident resulting in hepatic contusion and rib fractures. The patient developed right hypochondriac pain, jaundice, and gastrointestinal hemorrhage, consistent with Quincke's triad, suggesting pseudoaneurysm rupture. The diagnosis was confirmed by computed tomography (CT), and the patient underwent successful percutaneous embolization.

Despite established guidelines for managing blunt abdominal trauma, the approach to post-traumatic hepatic pseudoaneurysm remains controversial. While some advocate for prophylactic angiographic embolization to prevent bleeding, others favor conservative management due to the potential for spontaneous resolution. Currently, minimally invasive percutaneous embolization is the primary treatment modality. In cases where embolization fails, a transhepatic approach may serve as an alternative. The angioscan is a reliable diagnostic tool. Management is now predominantly minimally invasive, guided by radiological imaging. When conventional angiographic treatment fails, the transhepatic approach can be considered.

## Introduction

Pseudoaneurysm of the hepatic artery was first described in 1948 by Sandblom as a rare complication of blunt abdominal injury [1]. Post-traumatic hepatic pseudoaneurysm occurs in approximately 1.2% of patients with traumatic liver injuries [2]. Diagnosis is typically achieved using arteriography, contrast-enhanced computed tomography (CT angiography), or Doppler ultrasound [3]. Symptoms range from being clinically silent to presenting with rupture, resulting in intraperitoneal hemorrhage or rupture into the gastrointestinal tract, venous system, or biliary system. This report discusses the case of a 25-year-old patient with a hepatic artery pseudoaneurysm that presented several weeks after a blunt abdominal injury. This case adheres to the SCARE 2023 guidelines 2023 [4].

### Case Presentation

**Fig. 1:**
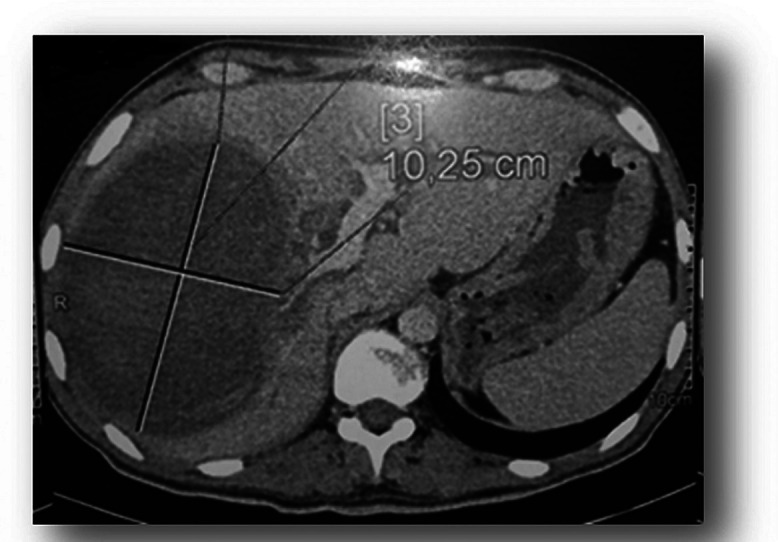
CT scan revealed an important focus of hepatic contusion occupying the IV segment of the liver.

A 25-year-old male with a history of Behçet's disease, managed with colchicine, was involved in a traffic accident. The trauma caused hepatic contusion and rib fractures (Fig. 1). Initial management included conservative treatment, as the patient was hemodynamically stable. He was hospitalized for one month.

On the 34th post-traumatic day, the patient presented with right hypochondriac pain and hematemesis. Physical examination revealed tenderness in the right hypochondrium, pallor, a heart rate of 88 bpm, and blood pressure of 120/70 mmHg. Laboratory tests indicated severe normocytic anemia with a hemoglobin level of 6 g/dL.

**Fig. 2:**
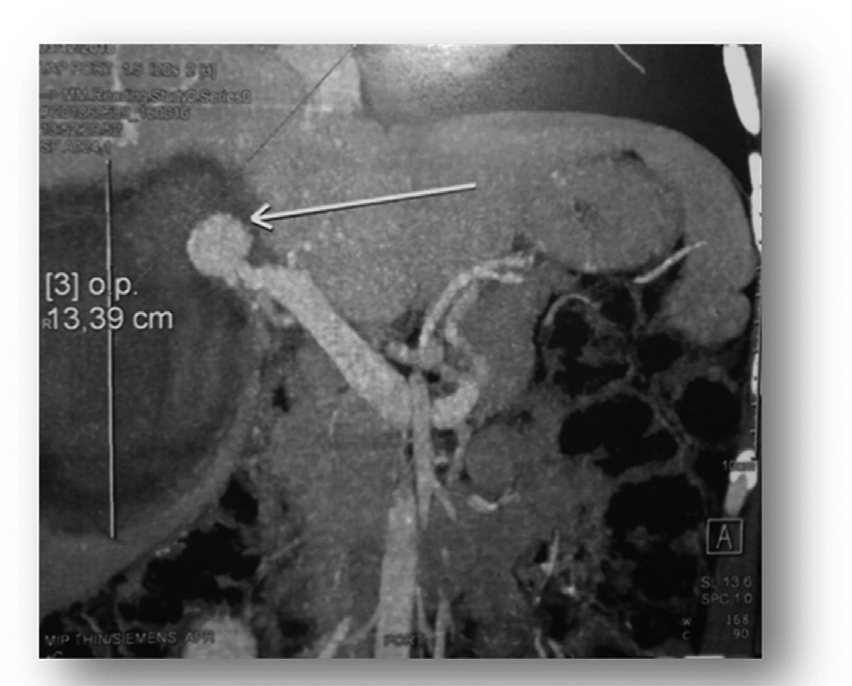
CT scan revealed an important focus of hepatic contusion occupying the IV segment of the liver with within it a spontaneously dense oval formation.

A CT scan (Fig. 2) revealed significant hepatic contusion in segment IV of the liver, with a spontaneously dense oval formation within the lesion. The gallbladder contained a dense collection. Intravenous contrast administration in the arterial phase revealed a hyperdense structure consistent with an aneurysmal sac arising from the right hepatic artery.

**Fig. 3:**
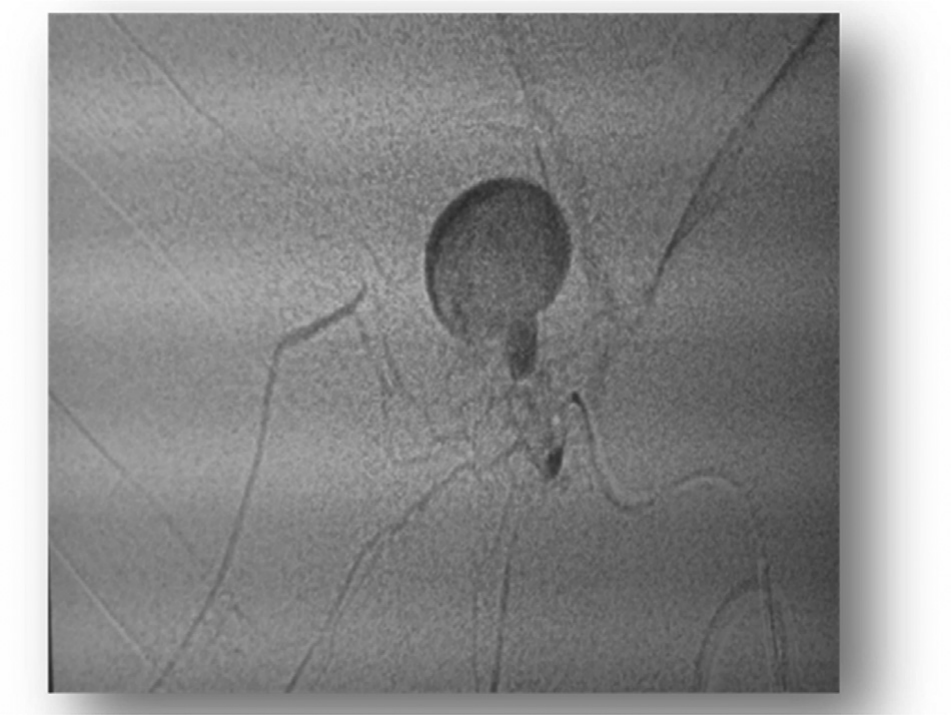
hyperdense formation indicative of an aneurysmal sac of the right branch of the hepatic artery.

Angiographic embolization was performed under fluoroscopic guidance. Embolization material was deployed into the aneurysmal sac, resulting in complete occlusion of the pseudoaneurysm while maintaining patency of the main hepatic artery and its branches (Fig. 3).

## Discussion

Conservative management is the standard of care for hemodynamically stable patients with blunt liver trauma, with success rates ranging from 85% to 94% [[3], [4], [5]]. delayed complications such as hemorrhage, pseudoaneurysm, abscess, hemobilia, and biliary complications can occur weeks to months after the initial trauma [2].

A pseudoaneurysm is a blood-filled cavity resulting from a vascular injury, retained by surrounding tissues rather than the vascular wall. This differentiates it from true aneurysms, which involve the vascular wall. Hepatic artery pseudoaneurysms are rare but can lead to life-threatening complications if ruptured.

Post-traumatic pseudo aneurysm of the hepatic artery is rare. Today, about 20 cases have been published [5]. Hepatic pseudo aneurysms occur either after hepatic trauma or after hepatic biliary surgery [3].

The typical progression of a pseudoaneurysm involves gradual enlargement with an associated risk of rupture into the bile ducts, portal system, jejunum, or peritoneal cavity. However, in the absence of complications, these pseudoaneurysms often remain asymptomatic. They are typically discovered incidentally or upon the appearance of clinical signs, including Quincke's triad (hepatic colic, jaundice, and hemobilia) first described in 1871 [3]. Quincke's triad is observed in less than 25% of cases but should strongly suggest the diagnosis.

The diagnosis of hepatic pseudoaneurysm often relies on imaging. CT angiography is a sensitive, noninvasive modality that allows visualization of vascular structures and associated complications. In our patient he allowed us to make the diagnosis. Magnetic resonance angiography provides high-resolution images and is useful for evaluating biliopancreatic anatomy. Angiography remains the gold standard for both diagnosis and therapeutic planning [3]

Management of pseudoaneurysms primarily involves minimally invasive embolization, which has supplanted surgical intervention. Embolization involves selective catheterization of the hepatic artery and deploying embolization material into the pseudoaneurysm sac. The embolization should be as selective as possible to decrease the risk of ischemia and the risk of collateral retrograde flow from distal branches to the point of embolization. A transhepatic approach may be considered in cases where endovascular techniques fail [6].

The mortality rate for ruptured pseudoaneurysms is approximately 20%, highlighting the importance of prompt diagnosis and timely intervention. Despite advances in interventional radiology, the recurrence rate for treated splanchnic aneurysms ranges from 1% to 15%, with reported failure rates varying between 5% and 25% depending on the series.

## Conclusion

Post-traumatic hepatic artery pseudoaneurysm is a rare but potentially fatal complication of abdominal trauma. Advances in imaging and minimally invasive techniques have improved outcomes. The angioscan remains a reliable diagnostic tool, and embolization is the treatment of choice. In cases of embolization failure, a transhepatic approach may be an effective alternative. This case contributes to the limited literature on this topic and underscores the importance of vigilant follow-up in patients with liver trauma.

## Prior presentation

Preliminary results were not presented.

## Clinical Trial Registration

None.

## Institutional review board/ethics committee approval

Not applicable.

## Institutional Review Board

Not applicable.

## Institutional Animal Care and Use Committee (IACUC)

Not applicable.

## Author contributions

Hiba Ben Hassine, Ahmed Hadj Tayeb collected and analyzed the data and drafted the original manuscript.

Hiba Ben Hassine, Maissa Jalleli, Sadek Ben Jabra, Jamel Saad,Faouzi Noomen designed this research, reviewed and edited the manuscript.

All authors read and approved the final manuscript.

## Data availability statement

The data that support the findings of this study are available on request from the corresponding author. All data is freely accessible.

## Disclaimer

The views expressed in this material are those of the authors, and do not reflect the official policy or position of the U.S. Government, the Department of Defense, or the Department of the Army.

## Institutional clearance

Institutional clearance does not apply.

## Patient consent

Written informed consent was obtained from the patient for publication of this case report and accompanying images. A copy of the written consent is available for review by the Editor-in-Chief of this journal on request.
